# Dealing With COVID-19 Patients: A Moderated Mediation Model of Exposure to Patients' Death and Mental Health of Italian Health Care Workers

**DOI:** 10.3389/fpsyg.2021.622415

**Published:** 2021-02-24

**Authors:** Igor Portoghese, Maura Galletta, Federico Meloni, Ilenia Piras, Gabriele Finco, Ernesto D'Aloja, Marcello Campagna

**Affiliations:** ^1^Department of Medical Sciences and Public Health, University of Cagliari, Cagliari, Italy; ^2^PhD School in Biomedical Sciences (Public Health), University of Sassari, Sassari, Italy; ^3^Emergency Department SS. Trinità Hospital, Azienda Tutela Salute Sardegna, Cagliari, Italy; ^4^Pain Therapy Service, University of Cagliari, Cagliari, Italy

**Keywords:** COVID-19 pandemic, healthcare workers, emotional requirements, rumination, intrusion symptoms, crying at work, patients' deaths

## Abstract

**Introduction:** The COVID-19 pandemic is asking health care workers (HCWs) to meet extraordinary challenges. In turn, HCWs were experiencing tremendous psycho-social crisis as they have had to deal with unexpected emotional requirements (ERs) arising from caring for suffering and dying patients on a daily basis. In that context, recent studies have highlighted how HCWs working during the COVID-19 outbreak manifested extreme emotional and behavioral reactions that may have impacted their mental health, increasing the risk for developing post-traumatic stress symptoms.

**Purpose:** The aim of the study was to investigate post-traumatic stress symptoms, such as intrusion symptoms, as a potential mediator of the link between ERs and crying at work, and whether rumination moderates the relationship between ERs and intrusion-based PTS symptoms among HCWs who have had to deal with patients dying from COVID-19.

**Methods:** An online cross-sectional study design was performed. A total of 543 Italian HCWs (physicians and nurses) participated in the study. Participation was voluntary and anonymous. We used the SPSS version of bootstrap-based PROCESS macro for testing the moderated mediation model.

**Results:** ERs had an indirect effect on crying at work through the mediating role of intrusion symptoms. Results from the moderated mediation model showed that rumination moderated the indirect effect of ERs on crying at work via intrusion symptoms, and this effect was significant only for high rumination. Furthermore, when we tested for an alternative model where rumination moderates the direct effect of ERs on crying at work, this moderation was not significant.

**Conclusions:** As the second wave of the COVID-19 pandemic is ongoing, there is an urgent need for decision-makers to rapidly implement interventions aimed at offering timely psychological support to HCWs, especially in those contexts where the risk of emotional labor associated to patients dying from COVID-19 is higher.

## Introduction

According to Kniffin et al. (2021), “the impacts of COVID-19 on workers and workplaces across the globe have been dramatic” (p. 2). The pandemic rapidly transformed normal work routines, forcing numerous organizations to move to virtual environments. However, a significant proportion of the workforce, such as health care workers (HCWs), continued in their daily routines as “essential professionals” (Kniffin et al., 2021) and had to meet unprecedented challenges. Around the world, HCWs have been highly celebrated as heroes by the popular media and national governments (Taylor et al., 2020), but since the mode of transmission of the COVID-19 was not clear in the early phases of this pandemic, they had to work in highly uncertain environments, exposing themselves to a higher risk of being infected.

In fact, from the moment the World Health Organization (World Health Organization, 2020) declared the Coronavirus Disease (COVID-19) outbreak to be a global pandemic, demands on health services were drastically increased, requiring HCWs to work beyond their limits. To tackle the pandemic effectively, HCWs had to be prepared in terms of knowledge, skills, and the emotional capability to cope with extraordinarily overwhelming negative emotions (Maunder et al., 2003; Lin et al., 2007; Son et al., 2019).

Globally, the first challenge for Health Care Systems was to reduce the risk of infection among HCWs. For this reason, the World Health Organization (2020), the Interim U.S. (2020), and The European Union information agency for occupational safety and health (EU-OSHA) (2020) published extensive guidelines aimed at protecting HCWs. In spite of this, a recent meta-analysis by Sahu et al. (2020) reported that during the first 3 months of the pandemic, ~10% of all COVID-19 patients were HCWs. The risk of infection among HCWs gradually decreased and brought under control as several critical measures were introduced in many health care systems. These included obligatory rules for mask wearing and social distancing measures for HCWs (Wang et al., 2020). Subsequently, as the infection risk for HCWs was brought under control, what rapidly emerged was the fundamental challenge to preserving mental health of HCWs. Unadkat and Farquhar (2020) suggested that “the paradox is that the more pressured things become, the more important it is to pay attention to the wellbeing of our staff.” In fact, an increasing number of studies have highlighted the tremendous psycho-social crisis HCWs were experiencing (Hu and Chen, 2020; Pfeferbaum and North, 2020) and the risk of a second pandemic concerning health and well-being of HCWs. Studies investigating the mental health of HCWs during previous pandemics (i.e., MERS and SARS) showed that these professionals were at high risk due to increased job demands, psychological distress, fatigue, and social stigma. Several recent studies have highlighted how HCWs working during the COVID-19 outbreak manifested fatigue, worries, frustration, isolation, depression, anxiety, stress, post-traumatic stress, and insomnia (Kang et al., 2020a,b). Additionally, in their systematic review and meta-analysis of the mental health of HCWs during the COVID-19 pandemic, Pappa et al. (2020) considered 13 studies, finding an overall anxiety incidence of 24.6%, an incidence of depression of 22.8%, and an insomnia incidence of 34.3%. In their review on psychological impact of epidemic and pandemic outbreaks, Preti et al. (2020) reported a prevalence of PTSD-like symptoms among HCWs of between 11 and 73.4%. The exposure to a traumatic or stressful event may result in post-traumatic stress symptoms that, in turn, may hinder HCWs' ability to cope with that experience. According to Raudenská et al. (2020), “the experience of a global pandemic like COVID-19 has the potential of being considered a mass traumatic event” (p. 555). There are three main PTS symptoms: (a) intrusive thoughts, which refer to the re-experiencing of the traumatic event; (b) avoidance, which refers to avoiding places/activities that can evoke intrusive memories; (c) and hyperarousal, which refers to symptoms of anger, irritability, hypervigilance, and difficulty concentrating (Horowitz et al., 1979; Weiss and Marmar, 1997). According to Ehlers et al. (2002), intrusive thoughts could be considered a core symptom of post-traumatic stress. Specifically, Taylor et al. (2020) suggested that COVID-related intrusive thoughts may be at the root of the COVID stress syndrome. In this sense, emotional distress in response to the COVID-19 pandemic may play an important role in exposing HCWs to PTS (Taylor et al., 2020). In fact, as COVID-19 compelled HCWs to deal with having to reassure suffering and dying patients on a daily basis, it took an extra emotional and psychological toll on them (Chevance et al., 2020). As reported on March 23, 2020, by Onder et al. (2020), the early case-fatality rate of patients dying from COVID-19 in Italy was 7.2%. During the early weeks of the pandemic, the clinical course of the COVID-19 was not yet clear, though there was high likelihood that patients would deteriorate rapidly into a critical condition or ultimately die (Chen et al., 2020). Globally, most health care systems were not prepared to manage a rapidly evolving pandemic. The sense of helplessness experienced by HCWs in seeing patients rapidly worsening and dying demanded huge emotional efforts on their part in offering psychological support to patients, such as exhibiting positive emotions and encouraging and sustaining suffering patients. Such strategies are common rules in many clinical contexts and are considered to be in-role job requirements (Diefendorff et al., 2006, 2011). However, these kinds of emotional requirements (ERs) have been shown to induce traumatic responses (Aghili and Arbabi, 2020; Cai et al., 2020), and it has been shown that dealing with traumatic events, such as providing lifeline services to patients with life threatening conditions, has led to HCWs manifesting PTS symptoms (Figley, 1995). In the large-scale emergency created by this pandemic, HCWs have had to deal with unforeseen emotional turmoil arising from both contact with patients and the pressure on themselves (Barello and Graffigna, 2020). HCWs have been exposed to extreme and severe conditions that have threatened their ability to cope, resulting in unusual and extreme emotional reactions (Meichenbaum, 1994).

In their narrative research, Daphna-Tekoah et al. (2020) investigated traumatic situations encountered by HCWs facing the COVID-19 pandemic. During their interviews, HCWs emphasized traumatic events related to patient's death and the high level of emotional intensity associated with it. Specifically, HCWs described “the pervading presence of death in the hospital, as particularly manifested in the agony of seeing people dying without their families beside them and in the procedures for preparing the deceased for burial by special, double wrapping of the dead body as a precaution against contagion” (Daphna-Tekoah et al., 2020, p. 7).

Pappa et al. (2020) reported that HCWs working in COVID-19 scenarios showed high rates of PTS symptoms, and these results were in line with previous studies during and after the MERS and SARS epidemics. For example, especially in the first phase of the COVID-19 pandemic, newspapers and social networks offered the first (indirect) picture of the psychological impact of this pandemic on HCWs. There were a number of stories reporting these dramatic experiences and extreme reactions (Maben and Bridges, 2020):

“*I broke down and cried today. I cried of exhaustion, of defeat. Because after 4 years of being an ER nurse, I suddenly feel like I know nothing*” (Sydni Lane, USA, Instagram and Facebook).

(Fick, 2020)

According to Lyon (2000), crying at work is among the most commonly reported behavioral manifestations of distress. It is considered to be an ineffective strategy for coping with personal difficulties to accomplish emotional labor (Soares, 2003), although many authors have reported that, in health care context, it is not uncommon that HCWs have cried at work due to being overwhelmed (Pongruengphant and Tyson, 2000; Wanzer et al., 2005). From an organizational and professional viewpoint, not crying at work in front of patients is cited among the emotion “display rules” health professionals should follow as it is seen as being professionally inappropriate. Hochschild (1983) has suggested that among the attributes required of caring, “emotional labor” requires HCWs to display positive emotions as part of their professional profile. In this sense, when HCWs are not able to cope with these ERs, there is a risk of developing distress. Also, pandemics are known to induce worries and rumination among HCWs, which, in turn, can trigger PTS (Bardeen et al., 2013; Boyraz and Legros, 2020).

Rumination is common after traumatic events (Watkins, 2008), and it has been hypothesized that in response to extraordinary continued or increased emotional distress, people may develop adaptative emotion-focused coping strategies, such as crying (Ehlers and Clark, 2000; Taku et al., 2008; Elwood et al., 2009). In the literature there are many different definitions of rumination (Siegle et al., 2004). In this context, in line with Cropley and Zijlstra's (2011) conceptualization of affective rumination, we define rumination as repetitive, intrusive thoughts with a negative focus, which includes post-event rumination (Jones et al., 2013). According to Conway et al. (2000), rumination on negative events “does not facilitate problem resolution, is a solitary activity, and is intrusive if the person is pursuing either self- or situationally imposed task-oriented goals” (p. 404). According to the effort-recovery theory (Meijman and Mulder, 1998), individuals invest mental and physical resources to deal with work-related demands. Rumination may activate a state of arousal, which may precipitate a depletion of resources and then inhibiting the recovery process (Brosschot et al., 2006). Kinnunen et al. (2019) showed that rumination may affect cardiovascular, autonomic, and endocrine nervous system activity, suggesting a pathogenic pathway to long-term disease outcomes (Ottaviani et al., 2016). In general, many authors have suggested that rumination may lead to a worsening of stressor–strain relationships (Jostmann et al., 2011; Jones et al., 2013). For example, the cognitive activation theory of stress (Ursin and Eriksen, 2010; Meurs and Perrewé, 2011) and the stressor-detachment model (Sonnentag and Fritz, 2015) emphasized that perseverative cognition such as ruminating (or psychological detachment) on job stressors may prolong workers' experience of stressful events. In this sense, rumination on the pandemic could be considered as sustained activation that may moderate the harmful effects of ERs on PTS symptoms.

**Figure 1:**
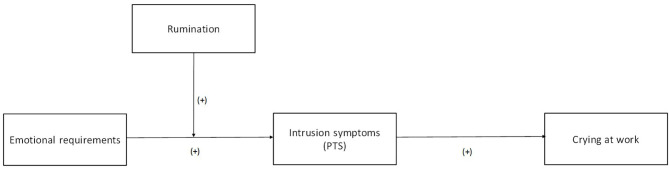
Hypothesized moderated mediation model.

The main purpose of this study was to examine whether PTS symptoms is a potential mediator of the link between ERs and crying at work, and whether rumination is a moderator of the link between ERs and PTS symptoms among HCWs who have had to deal with patients dying from COVID-19 (Figure 1):

Hypothesis 1: the relationship between emotional requirements and crying at work will be mediated by PTS symptoms.

Hypothesis 2: rumination on the pandemic will moderate the strength of the relationship emotional requirements and crying at work will be mediated by PTS symptoms, such that the mediated relationship is stronger under high rumination than under low rumination.

## Method

### Data Collection

An online survey was conducted using LimeSurvey and disseminated by sharing the link of the survey through social networking platforms. The inclusion criteria were as follows: (1) being a nurse or physician in Italy, and (2) having worked with patients who died from COVID-19. The link contained an invitation to join an online survey entitled “Health professionals and the Coronavirus SARS-COV-2 pandemics: an explorative study.” On the first page, we described the study's objectives, the time necessary to complete the survey (<10 min), the inclusion criteria, and the ethical issues behind the study. Participants received information about their involvement in the study together with a guarantee (1) that it was voluntary, (2) that it was anonymous, and (3) that no information that could identify respondents would be collected. Only individuals who agreed to the study's conditions completed the survey. The survey was available online from March 27 to May 31, 2020.

Based on previous studies of PTS symptoms and distress, an effect size of *f* ^2^ = 0.15 (*R*^2^ = 0.30) was expected in this study. Assuming an alpha level of 0.05, using a two-tailed test for multiple regression random effects model, seven predictors, and a desired power of 0.95, a power analysis using G^*^Power 3.1.9 (Faul et al., 2009) indicated that a minimum sample size of 89 participants was required.

### Measures

We measured ERs, mainly displaying positive emotions (“Reassuring patients who are distressed or upset”), by adapting one item from the Emotion Work Requirements (Best et al., 1997). PTS symptoms were measured by using the intrusion subscale (two items; inter-item correlation = 0.82) from the Italian validation of the brief Impact of Event Scale (IES-6; Horowitz et al., 1979; Thoresen et al., 2010; Giorgi et al., 2015). Rumination about the pandemic was measured by adapting two items (inter-item correlation = 0.76) from the Rumination on Sadness Scale (Conway et al., 2000). Finally, we measured the frequency of crying at work due to the difficulty in handling the situation by adapting one item (“I have been crying at work because I felt like I could not take it anymore”). Items were answered on a five-point Likert scale from one (strongly disagree) to five (strongly agree).

#### Control Variables

In order to lessen problems related to spurious relationships, age, sex, working region, tenure, and number of patients who had died from COVID-19 were statistically controlled in hypotheses testing. Recently, Williams and Williams (2020) suggested that personal and occupational characteristics may represent important risk factors in developing PTS symptoms. Specifically, Williams and Williams (2020) reported that younger age, being male, and a high job tenure appear to lower workers' risk of developing PTS. Furthermore, many scholars suggested that women appear more likely than men to engage in behavioral expression of emotions, such as crying (Nolen-Hoeksema and Jackson, 2001). Finally, as in the first wave of the pandemic where 70.2% of positive cases and 79.4% of deaths occurred in Northern Italy (Goumenou et al., 2020), we considered working region and number of patients who had died from COVID-19 (1 = 1 patient, 2 = 2–5 patients, 3 = 6–10 patients, 4 = more than 10 patients) as control variables in rumination and ERs.

### Data Analyses

We tested our hypotheses using path analytic procedures (Preacher et al., 2007) and conducted bootstrapping analysis to assess the significance of both mediation and moderated mediation models (Shrout and Bolger, 2002; Hayes, 2015). We used the SPSS version of Hayes' (2018) bootstrap-based PROCESS macro for testing the multiple mediation model (release 3.5). Specifically, mediation and moderated mediation analyses were performed using models 4 and 7, respectively, in the PROCESS macro for SPSS developed by Hayes (2013, 2017).

We used the bootstrap confidence intervals (CIs) to determine the significance of the effects based on 5000 random samples (Hayes, 2013). When the CIs do not include zero, then the effect is significant. All variables were mean-centered in the mediating and moderating analyses. Simple slope analysis was carried out to examine the nature of the moderation effect.

## Results

A total of 2759 Italian HCWs agreed to participate in the survey with 1621 (59%) completing the survey (answering all items in the survey). As our main inclusion criteria were being nurses/physicians in Italy and working with patients who died from COVID-19, a total of 543 were included in the study.

The study population consisted of 353 (65.0%) females and 190 (35.0%) males. Participants ranged from 22 to 71 years of age, *M* = 42.87, *SD* = 10.94. Concerning profession, 381 (70%) were nurses and 162 (30%) were physicians. Regarding working region, 329 (60.59%) reported working in Northern Italy (Piemonte, Liguria, Lombardia, Trentino-Alto Adige, Veneto, Friuli-Venezia Giulia, and Emilia-Romagna). Concerning patient's death, 113 (20.81%) reported that one of their patients had died from COVID-19, 202 (37.20%) reported that from two to five patients had died from COVID-19, 94 (17.31%) reported that six to 10 patients had died from COVID-19, and 134 (24.68%) reported that more than 10 patients had died from COVID-19.

Means, standard deviations, kurtosis, skewness, and intercorrelations between all variables are presented in Table 1.

**Table 1:** Means, standard deviations, kurtosis, skewness, and Pearson's correlations among variables.

		**M**	**SD**	**Kurtosis**	**Skew**	**1**	**2**	**3**
1	Emotional requirements	3.96	1.05	0.85	−1.12	-		
2	Intrusion symptoms (PTS)	3.02	0.79	−0.04	−0.40	0.21^*^	-	
3	Rumination	3.37	1.03	−0.57	−0.39	0.12^*^	0.44^*^	-
4	Crying at work	2.36	1.37	−1.05	0.55	0.24^*^	0.44^*^	0.35^*^

*PTS, Post-Traumatic Stress; M, mean; SD, standard deviation*.

*N = 543*.

* *p < 0.01*.

### Direct and Indirect Effects

To test the hypothesis that ERs have an indirect association with crying at work as a result of intrusion symptoms, we conducted a simple mediation analysis in line with the procedures presented by bib30 (2017; model 4). Bootstrapping was set to 5,000 resamples. After controlling for age, sex, working region, tenure, and number of patients who had died from COVID-19, we found significant indirect [β = 0.11, BootSE = 0.02, 95% Boot CI (0.06, 0.16), *p* < 0.001] and direct [β = 0.19, BootSE = 0.05, 95% Boot CI (0.09, 0.30), *p* < 0.001] effects of ERs on crying at work. Therefore, these results partially confirmed an indirect effect of ERs on crying at work through the mediating role of intrusion symptoms. This model explained 19% of variance in crying at work (Table 2). Concerning control variables entered into the model, age, sex, working region, tenure, and number of patients who had died from COVID-19 explained 1% of variance in crying at work.

**Table 2:** Test of the mediational model.

	**Intrusion Symptoms (PTS)**			**Crying at Work**		
**Predictor**	**β**	**BootSE**	**95% Boot CI (LL;UL)**	**β**	**BootSE**	**95% Boot CI (LL;UL)**
Constant	1.42	0.51	(0.43;2.42)	−0.61	0.74	(−2.07;0.85)
Age	0.01	0.01	(−0.001;0.02)	0.00	0.01	(−0.01;0.02)
Sex	0.13	0.08	(−0.02;0.28)	0.15	0.11	(−0.07;0.37)
Tenure	−0.001	0.01	(−0.01;0.01)	0.01	0.01	(−0.01;0.02)
Working region	−0.01	0.01	(−0.03;0.01)	−0.02	0.01	(−0.05;0.002)
NPD COVID-19	0.16^*^	0.04	(0.09;0.23)	0.03	0.05	(−0.07;0.13)
ERs	0.20^*^	0.04	(0.13;0.27)	0.19^*^	0.05	(0.09;0.30)
Intrusion symptoms (PTS)				0.52^*^	0.06	(0.40;0.65)
*R*^2^	0.12			0.19		
Δ*R*^2^				0.07		

*BootSE, bootstrapped standard error; Boot CI, bootstrapped confidence interval; NPD COVID-19, Number of patients who had died from COVID-19*.

* *p < 0.001*.

### Tests of Moderated Mediation

Next, we tested for moderated mediation (Table 3) where rumination moderates the indirect effect of ERs on crying at work via intrusion symptoms (PROCESS model 7; Preacher et al., 2007; Hayes, 2017). Specifically, rumination moderated the indirect effect from ERs on crying at work via intrusion symptoms [β = 0.07, BootSE = 0.03, 95% Boot CI (0.01, 0.13), *p* < 0.05]. Furthermore, as we found that ERs had a direct effect on crying at work, we tested for an alternative model (PROCESS model 8; Preacher et al., 2007; Hayes, 2017) where rumination is supposed to moderate the direct effect of ERs on crying at work. Results showed that this moderation was not significant [β = 0.06, BootSE = 0.05, 95% Boot CI (−0.03, 0.15), *p* > 0.05]. Furthermore, our results were confirmed by the significant index of moderated mediation [β = 0.03, BootSE = 0.01, 95% Boot CI (0.002, 0.056), *p* < 0.001], which suggested that the indirect effect of ERs on crying at work was linearly related to rumination (Hayes, 2015). This moderated mediational model explained 23% of variance in intrusion symptoms and 24% in crying at work. Concerning control variables entered into the model, age, sex, working region, tenure, and number of patients who had died from COVID-19 explained 3.5% of variance in intrusion symptoms and 0.5% in crying at work.

**Table 3:** Test of the moderated mediational model.

	**Intrusion Symptoms (PTS) (Model 7)**			**Crying at Work (Model 8)**		
**Predictor**	**β**	**BootSE**	**95% Boot CI (LL;UL)**	**β**	**BootSE**	**95% Boot CI (LL;UL)**
Constant	2.16	0.46	(1.26;3.06)	0.42	0.7	(−0.96;1.80)
Age	0.01	0.00	(−0.00;0.02)	0.00	0.01	(−0.01;0.001)
Sex	0.13	0.07	(−0.01;0.28)	0.15	0.11	(−0.07;0.37)
Tenure	0.00	0.01	(−0.01;0.01)	0.01	0.01	(−0.01;0,02)
Working region	−0.01	0.01	(−0.02;0.01)	−0.02	0.01	(−0.05;0.001)
NPD COVID-19	0.12^**^	0.03	(0.06;0.19)	0.03	0.05	(−0.07;0.13)
ERs	0.18^**^	0.03	(0.11;0.25)	0.19^**^	0.05	(0.09;0.30)
Rumination	0.29^**^	0.03	(0.22;0.35)	0.32^**^	0.05	(0.22;0.43)
Intrusion symptoms				0.38^**^	0.07	(0.25;0.51)
ERs × Rumination	0.07^*^	0.03	(0.01, 0.13)	0.06	0.05	(−0.03;0.15)
*R*^2^	0.23			0.24		
Δ*R*^2^				0.01		

*BootSE, bootstrapped standard error; Boot CI, bootstrapped confidence interval; NPD COVID-19, Number of patients who had died from COVID-19*.

*N = 543. Unstandardized regression coefficients are reported. Bootstrap sample size = 5000*.

* *p < 0.05*,

** *p < 0.001*.

As shown in Table 4, the examination of the conditional effect of ERs on crying at work at low (−1 SD) and high (+1 SD) rumination revealed that this effect was significant only for high rumination [β = 0.13, SE = 0.03, 95% Boot CI (0.07, 0.20), *p* < 0.001].

**Table 4:** Estimates and bias-corrected bootstrapped 95% confidence intervals.

	**Levels of Rumination**	**β (BootSE)**	**95% Boot CI (LL;UL)**
Direct effect		0.19 (0.05)	(0.089;0.294)
Indirect effect	−1 SD	0.05 (0.03)	(−0.013;0.108)
	+1 SD	0.13 (0.03)	(0.071;0.195)

*Conditional indirect effect of ERs on crying at work at values of the rumination (model 7)*.

*BootSE, bootstrapped standard error; Boot CI, bootstrapped confidence interval; LL, lower 95% level confidence interval; UL, upper 95% level confidence interval*.

**Figure 2:**
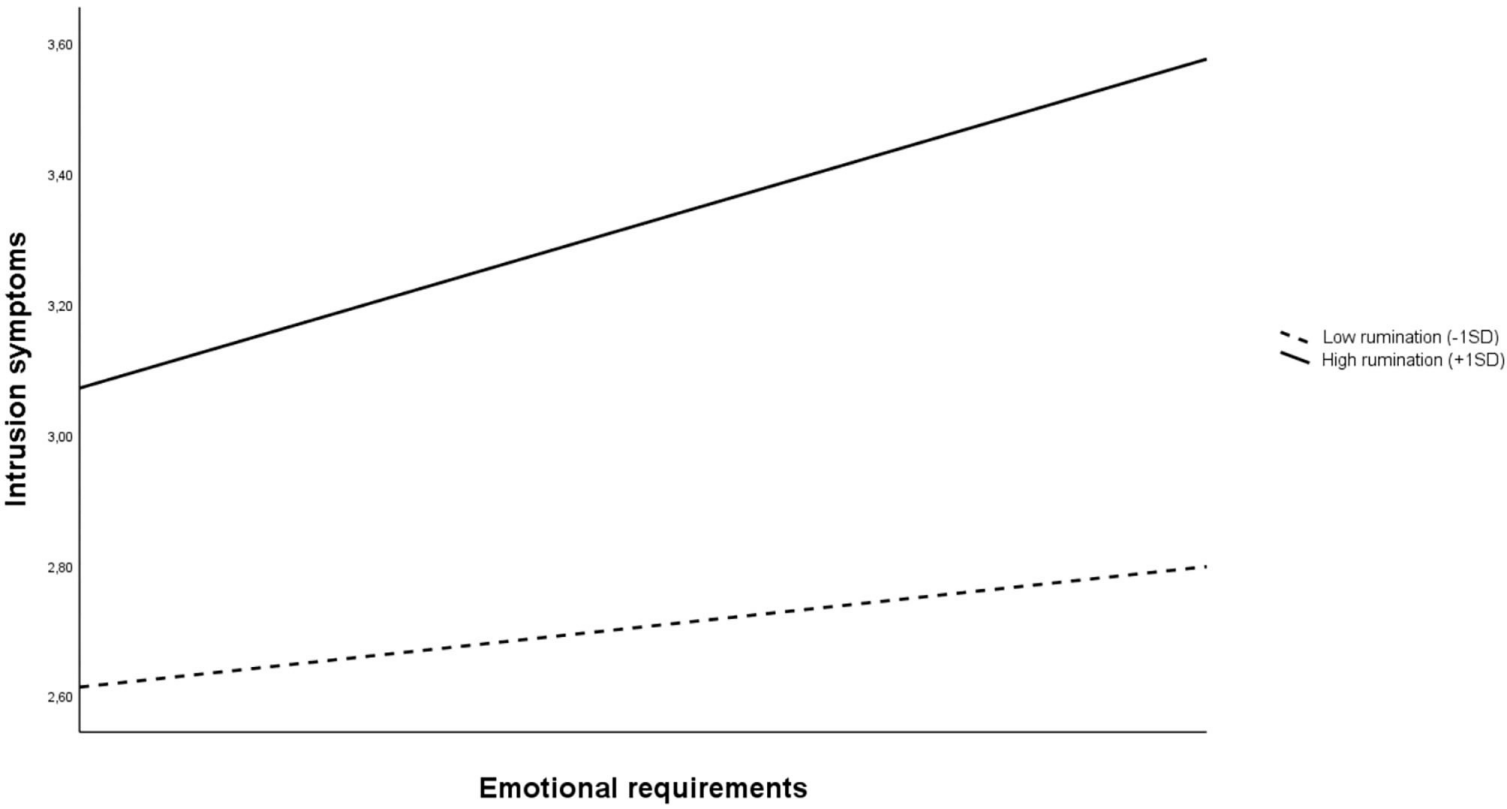
Plot of the relationship between ERs and intrusion symptoms at low (−1 SD) and high (+1 SD) rumination.

Finally, we performed the simple slope analysis, plotting the relation between ERs and intrusion symptoms in HCWs at low (−1 SD) and high (+1 SD) rumination in Figure 2. When rumination was low, the relationship between ERs and intrusion symptoms was significant [β = 0.14, BootSE =0.06, 95% Boot CI (0.19, 0.27)]. This relationship was significantly stronger among HCWs with high rumination [β = 0.27, BootSE = 0.07, 95% Boot CI (0.13, 0.45)].

## Discussion

Patient death can be an emotionally stressful event that may increase the risk of experiencing mental health problems (Harder et al., 2020). Our study was aimed to investigate the experiences of HCWs during the first 2 months of the COVID-19 pandemic in Italy. Specifically, we analyzed whether intrusion symptoms mediated the relationship between ERs and crying at work, and whether rumination about the pandemic moderated the relationship between ERs and intrusion symptoms among HCWs who have had to deal with patients dying from COVID-19. Specifically, our findings suggested that, the emotional involvement in reassuring patients who were distressed or upset for their health, as well as seeing patients dying without their families beside them (Daphna-Tekoah et al., 2020), had the potential to trigger trauma and thus increase the risk of developing intrusion symptoms. This is in line with previous studies that showed how exposure to patient-related stressful situations makes HCWs susceptible to PTS (Kerasiotis and Motta, 2004; Adriaenssens et al., 2012; de Wijn and van der Doef, 2020; Portoghese et al., 2020). Furthermore, our results confirmed that, during pandemics, HCWs are exposed to different kinds of job and emotional demands that may reduce their well-being and expose them to intrusion thoughts (PTS symptom) (Aghili and Arbabi, 2020; Blanco-Donoso et al., 2020; Cai et al., 2020; Chevance et al., 2020; Daphna-Tekoah et al., 2020; Pappa et al., 2020).

Among outcomes relating to intrusion symptoms, we considered an emotional reaction such as crying at work due to the difficulty of handling situations at work. Specifically, we observed that intrusion symptoms were a significant predictor of crying at work and that it significantly mediated the relationship between ERs and crying at work. However, we found that ERs had a significant effect on crying at work. Therefore, since we considered crying at work to be an indication of great difficulty in dealing with emotional labor (Figley, 1995), it is an important sign of emotional distress that should be considered. In general, our findings supported previous studies that showed how emotional demands are an antecedent of PTS symptoms (Park et al., 2018) and how HCWs may develop adaptive emotion-focused coping strategies, such as crying (Ehlers and Clark, 2000; Taku et al., 2008; Elwood et al., 2009) in response to emotional distress generated by the pandemic.

Furthermore, the present study highlights the role of rumination (on the pandemic) as a moderator of the relationship between ERs and intrusion. To our knowledge, the moderating role of rumination is still understudied in pandemic literature, and it suggested that rumination about the pandemic may exacerbate the effect of ERs on PTS symptoms. Specifically, we found that among HCWs exposed to patient death, the relationship between ERs and intrusion symptoms was stronger when rumination was higher. Our results are in line with the general assumption that rumination plays a significant role in worsening stressor–strain relationship (Takano et al., 2011; Jones et al., 2013). According to Lee (2019), rumination has been considered as a maladaptive coping strategy to traumatic events that may be a significant risk factor for PTS. In particular, our results should be considered in light of the stressor-detachment model proposed by Sonnentag and Fritz (2015) where rumination is considered as a concept that overlaps with lack of psychological detachment. In this sense, the less HCWs show psychological detachment (high rumination), the stronger is the relationship between stressors and intrusive symptoms.

Our study has important practical implications for mental health of HWCs in this pandemic context. During the early weeks of the COVID-19 pandemic, Greenberg (2020) suggested that “it is imperative that managers of [health-care workers] take measures to protect the mental health of staff.” According to a recent meta-analysis, trauma-exposed organizations, such as health care organizations, should provide rapid support to their staff, implementing early post-trauma interventions. Until the pandemic ceases, health care organizations should consider to implement early interventions based on psychological debriefing that are aimed “to prevent the development of adverse reactions” before they arise (Richins et al., 2020). Among these interventions, there are (1) debriefing within a group setting, (2) focusing on narrative construction, and (3) social cohesion to support post-incident recovery. In their meta-analysis, Richins et al. (2020) found that those interventions were linked to reduced PTSD symptom severity. Furthermore, leaders play a crucial role in the implementation of these early interventions. In fact, Mitchell and Stevenson showed that when supervisors show support to the staff, it reduced the likelihood of psychological problems (Mitchell and Stevenson, 2000).

Recently, Chen et al. (2020) investigated mental health of medical staff in China during the COVID-19 outbreak, suggesting that it was crucial for health care systems providing timely personalized support through hotline teams, media, or multidisciplinary teams. However, their study highlighted that “the implementation of psychological intervention services encountered obstacles, as medical staff were reluctant to participate in the group or individual psychology interventions provided to them” (p. e15). Accordingly, adopting a bottom-up approach helped in adjusting and tailoring specific interventions aimed to satisfy specific staff's needs. Among those needs, the medical staff requested specific training on psychological skills to deal with emotional demands. As the high likelihood of a second wave of COVID-19 in autumn, health care managers should consider to rapidly implement interventions to strengthen staff's resilience. In fact, in literature, there is evidence that those interventions showed positive effects in the immediate or short term (Delgado et al., 2017).

Finally, Ornell et al. (2020) argued that it would be crucial that organizational interventions should be aimed to offer coping strategies to deal with intrusive thoughts. Furthermore, they suggested that hospital managers should promote emotional interventions aimed to “facilitate intra-team support, empathy, and compassion toward more fragile colleagues” (p. 4).

Some limitations of the present study should be addressed. The first concerns the generalizability of our results as we used an online cross-sectional study from a convenience sample. The impact of this pandemic on HCWs' mental health should be investigated across time. Thus, future studies should consider longitudinal data to overcome cross-sectional limitations. Secondly, we assessed ERs and crying at work using a single-item measure for both. As we began collecting data in the middle of the first COVID-19 wave in Italy (March 27), we followed a practical criterion, keeping our survey short (< 10 min). However, single-item measures are very common in occupational health psychology and epidemiological studies, and there is general agreement that they are valid and reliable (Fisher et al., 2016). Future research should consider the use of reliable multi-item measures. Third, we investigated only intrusion symptoms, neglecting avoidance, and hyperarousal symptoms. Future research should consider measuring all PTS symptoms and their relationship in the proposed model. Fourth, the frequency of crying at work asking participants to indicate to what extent they agree or disagree with the statements. Future research should consider measuring the frequency of crying adopting a daily perspective as it would be possible that we were not able to assess if participants cried many times per day. Fifth, we did not measure health status of participants, such as depression, anxiety, or other health-related quality of life indicators. In this sense, future research should consider using both valid clinical measure of health status and self-rated health measures. Finally, we did not consider any personal or organizational resource in our model. Further research is necessary for understanding how HCWs' personal/organizational resources, such as resilience, self-efficacy, and peer/supervisor support, could moderate/buffer the negative impact of ERs and rumination and, eventually, facilitate post-traumatic growth.

## Conclusions

As a third wave of the COVID-19 pandemic represents an imminent global risk, government and hospital management should consider to rapidly implement regional and national interventions for protecting HCWs' well-being. The lessons learned from this pandemic should help decision-makers to promote readiness in offering timely psychological support to HCWs treating patients with COVID-19. In this phase, it is crucial that decision-makers developed awareness of the impact of this pandemic on the HCWs' mental health. Inefficacious and/or late interventions may represent a point of no return for many health care work force.

## Data Availability Statement

The raw data pertaining to analyses performed in this study are available from the authors upon reasonable request.

## Ethics Statement

Ethical review and approval was not required for the study on human participants in accordance with the local legislation and institutional requirements. This study was developed in accordance with the ethical standards of the institutional and/or national research committee and with the 1964 Helsinki declaration and its later amendments. No treatments or false feedbacks were given, and no potential harmful evaluation methods were used. Participation was completely voluntary, and participants could drop out at any time without any negative consequences. All data were stored only using an anonymous ID for each participant. Written online informed consent to participate in the survey was obtained by clicking on “I accept”.

## Author Contributions

IPo, MG, FM, and IPi designed the study, developed the survey, and managed the online survey and the data. IPo wrote the methods. IPi and FM helped to prepare the references and helped with first draft of the manuscript. IPo and MG supervised the analysis. FM, IPi, ED'A, GF, and MC revised the final version of the manuscript. All authors read and approved the final version of the manuscript.

## Conflict of Interest

The authors declare that the research was conducted in the absence of any commercial or financial relationships that could be construed as a potential conflict of interest.
